# Development of a prognostic model for 1-year survival after fragile hip fracture in Chinese

**DOI:** 10.1186/s13018-021-02774-y

**Published:** 2021-11-27

**Authors:** Hairui Fu, Bin Liang, Wei Qin, Xiaoxiong Qiao, Qiang Liu

**Affiliations:** 1 Department of Orthopedics, Third Hospital of Shanxi Medical University, Taiyuan, 030032 China; 2 Department of Orthopedics, Affiliated Fenyang Hospital of Shanxi Medical University, Fenyang, 032200 China; 3 Department of Medical Record Management, Affiliated Fenyang Hospital of Shanxi Medical University, Fenyang, 032200 China; 4 Information Center, Affiliated Fenyang Hospital of Shanxi Medical University, Fenyang, 032200 China

**Keywords:** Prognostic model, Development, Fragile hip fracture, Survival

## Abstract

**Background:**

No prognostic model for the survival of fragile hip fracture has been developed for Asians. The goal of this study was to develop a simple and practical prognostic model to predict survival within 1 year after fragile hip fracture in Asians.

**Methods:**

A single-center retrospective cohort study was designed. Under a multivariable Cox proportional hazards regression model, we used the preoperative characteristics of patients to predict survival within 1 year after hip fracture. We built a full model and then used the least absolute shrinkage and selection operator (LASSO) method to further shrink the model coefficients and achieved variable screening. Finally, we obtained a LASSO model. The model performance was evaluated with Nagelkerke’s R^2^ and the concordance (c) statistic. We assessed the internal validity with a bootstrapping procedure of 1 000 repetitions.

**Results:**

A total of 735 eligible patients were admitted to our department for hip fracture from January 2015 to December 2020, but 11 (1.5%) patients were lost to follow-up. Among the remaining patients, 68 (9.3%) died within 1 year after hip fracture. We identified 12 candidate predictors from the preoperative characteristics of the patients. The last model contained nine predictors: surgery, age, albumin, sex, serum creatinine, malignancy, hypertension, ability to live independently, and cardiovascular and cerebrovascular diseases. Among them, surgery, age, and albumin are effective predictors of survival. The discrimination c statistic of the model is 0.814 (95% confidence interval 0.762–0.865); the corrected value through internal validation is 0.795.

**Conclusions:**

This prognostic model can accurately predict a 1-year survival rate for patients with fragile hip fractures. This information can help clinicians develop a reasonable and personalized treatment plan.

## Introduction

### Background and objectives

Fragile hip fractures mainly occur in middle- and old-aged patients, especially elderly patients. These patients tend to have more medical complications. It is not uncommon for them to die shortly after discharge or even during hospitalization. This poses a challenge for orthopedic surgeons and patients when choosing treatment options. For example, for patients who die shortly after surgery, their family members may think that the doctor's recommendation to choose surgery is wrong; conservative treatment may have been more appropriate, whether in terms of economic benefits or survival time. How to use the preoperative characteristics of patients to quickly distinguish the high-risk patients who will die shortly after a hip fracture from the low-risk patients and thus inform the doctors and family members about the patient’s prognosis in advance is of great practical significance for both providing personalized treatment and alleviating doctor–patient conflicts.

The existing prognostic models [1–7] have many problems. First, some models use other models as predictive factors. This makes the model complex and difficult to understand. It inevitably reduces their practicality [4–6]. For example, one model contained only six predictors, three of which are other models [5].

Second, some models use outcome variables as predictors, such as postoperative complications. This greatly limits their applicability [1]. Furthermore, there are methodological shortcomings: improper selection of the statistical model, for instance, studying survival outcomes with a logistic regression model [5]; studying binary outcomes with a linear regression model [2]; converting all continuous variables into categorical variables [3, 6], resulting in lower information utilization; not correcting for overfitting [6]; and selecting all candidate predictors with univariate analysis [6]. Finally, no similar model has been developed for Asians.

The goal of this study was to develop a simple and practical prognostic model for Asians using simple and easy-to-obtain preoperative characteristics to predict survival within 1 year after fragile hip fracture (SFHF). This model is mainly aimed at clinicians. They can quickly predict the risk of death of patients based on this model before surgery. This provides a reference for both doctors and patients to jointly develop reasonable personalized treatment plans.

The basic principle of useful model development is that practicality takes precedence over accuracy.

## Methods

The report for this paper follows the TRIPOD Statement [8].

### Source of data

The data came from a retrospective cohort study designed specifically for the development of this model. The study was conducted at Fenyang Hospital, a general university hospital in Shanxi Province, China. The participants in the model were patients who had been admitted consecutively to the orthopedics department of the hospital for hip fractures from January 2015 to December 2020. The follow-up started on April 13, 2021, and ended on May 28, 2021.

### Participants

Our hospital is a municipal tertiary hospital, and its patients mainly come from surrounding counties and cities. The inclusion criteria were: 1. Age ≥ 50 years . The author chose 50 as the cutoff point to include as many fragile hip fracture patients as possible, especially for some women whose fragile fractures occur quite early. 2. Fragile fracture. Fragile fractures or low-energy fractures (considered synonyms here) refer to hip fractures that occur when patients fall from a standing height or lower, for example, falling when walking or when standing up or sitting down. High-energy fractures such as high falls, traffic accidents, high-energy injuries, and fights were excluded. Hip fractures in this study include femoral neck and intertrochanteric and subtrochanteric fractures. Periprosthetic and pathological fractures were excluded.

In our hospital, the basic process after a patient is admitted is: They are considered for surgical treatment initially and complete the routine preoperative tests such as necessary laboratory tests, fracture sites, and chest X-rays. If the patient has a medical disease, we invite the relevant departments for consultation and give symptomatic treatment. With the consent of the patient, surgery is performed as soon as possible after stabilizing the patient's general condition. Such a scheme results in very few patients undergoing surgery within 24 h of hospitalization. Conservative treatment should be adopted if the doctor believes that the patient cannot tolerate surgery or if the patient refuses the procedure.

The general choice of surgical treatment methods is femoral neck fracture-3 hollow nail fixation (< 65 years old) or half hip or total hip replacement (≥ 65 years old) and intertrochanteric or subtrochanteric fracture-proximal femur intramedullary nail fixation.

### Outcome

The outcome of interest was any cause of death within 1 year after hip fracture. It was determined through telephone interviews. All interviews were conducted after the patient data had been collected.

To ensure a high successful follow-up rate, we made the following efforts.

**Fig. 1:**
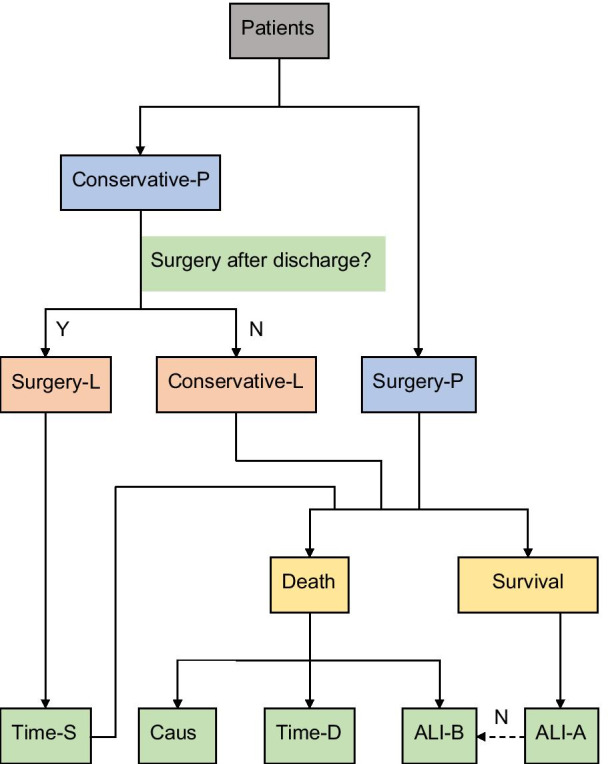
Telephone interview flow chart. (*Note*: -P: primary; -L: last; -S: surgery; -D: death; Y: yes; N: no; dotted arrow: performed only if ALI-A be no)

1. We developed the principle of telephone interviews by minimizing the content of the interviews as much as possible. The interview content varied according to the condition of the patient. In addition to collecting death and time information, we collected the cause of death and the patient's self-care ability before the fracture and/or after recovery. The purpose was to make the interview easier to accept by the family. According to the treatment methods in our hospital, patients were divided into two categories: surgical and conservative. For the former, if the patient died, we asked about the time of death, the reason, and the self-care situation before the fracture; if the patient survived, we asked about the current self-care situation, whether the patient could take care of himself/herself now, and then ended the interview. For conservatively treated patients, we continued to ask about the self-care situation before the fracture. In this group, they could not simply be classified as conservative treatment because they might go to other places for surgery after leaving our hospital. Therefore, we first had to determine whether they had been following conservative treatment before proceeding with the interview. If not, they were classified as surgical patients and were interviewed like surgery patients in our hospital in addition to questions about the timing of the operation. In this way, at least one question was asked in each interview. For example, those who had recovered their self-care ability after surgery could end the interview after just asking about their current recovery status. At most five questions were asked. For example, among those who died after going to other hospitals for surgery, ⑴ whether they were operated on after discharge, ⑵ the operation time, ⑶ the time of death, ⑷ the cause, and ⑸ their ability of self-care before the fracture (Fig. 1).
2. We set up time reference points to help the patients' families recall when death had occurred. For example, we ask the family members of the patients who had surgery after discharge, "Which day after the discharge or fracture was the surgery was performed." For the patients who had died but their family members could not recall the specific time of death, we used a method of narrowing the time range gradually. For example, "In which season did the death occur? Before or after the Spring Festival? Did it occur after the Lantern Festival?"
3. In cases of an incorrect telephone call, the family could not determine the specific time of death, or unwillingness to cooperate, we returned to the original medical record, checked the telephone number, or looked for other telephone numbers that had been recorded and then attempted a re-interview.
4. When the original medical records were checked and the interviews were still unsuccessful, we turned to the community medical system, which registered the telephone numbers of all families living in the community. If we were able to obtain a new phone number, then we interviewed them again.

Through the above methods, most of the patients lost to follow-up could successfully be contacted. The rest were regarded as missing data.

### Predictors

Based on the principle of modeling (practicality is preferred to accuracy) and the purpose (to provide a reference for formulating a treatment plan), we determined the principle of selecting candidate predictors: 1. Simplicity. Predictors were selected from the patient's medical history and the routine preoperative preparations. 2. Stability. We measured predictors relatively stable across different testers or patients. 3. Independence. Predictors that could be determined independently by an orthopedic surgeon without consultation with other departments should be preferred. 4. Rapidity. The results of the predictors need to be determined quickly within 24–48 h after admission, without long waits. 5. Quantitative indicators are prioritized over qualitative indicators. 6. Relevance. Select relevant factors that affect the implementation of surgical treatment. 7. Subject matter knowledge. Select the indicators that have been shown to be relevant to survival rates.

Initially, 24 indicators were extracted for each patient to provide detailed sample characteristics, including their general characteristics: medical insurance, age, and sex; disease characteristics: fracture site, fracture type, time from the fracture to hospitalization, time from the hospitalization to surgery, and length of stay (LOS); medical history: diabetes, hypertension (HYP), malignancy (MAL), kidney disease (KD), and lung disease (LD) on admission; the ability to live independently (ALI) before the fracture; cardiovascular and cerebrovascular diseases (CCD); test results (all using the first test value after admission): partial pressure of oxygen (PaO_2_), fasting blood sugar (BS), serum creatinine (SC), hemoglobin (Hb), total protein (TP), albumin (ALB), and mean arterial pressure (MAP); and treatment: skeletal traction or surgery. To understand the impact of surgery (SUR) on survival, SUR was deliberately used as a predictor, although it was not a preoperative indicator.

Some of the indicators were defined as follows: 1. Medical insurance: This includes employee medical insurance and nonemployee medical insurance. China has achieved full medical insurance coverage since 2011. Nonemployee medical insurance mainly includes new rural cooperative medical insurance, a small number of other types of commercial insurance, and self-paid medical treatment. The type of medication can reflect the economic situation of a patient. 2. Fracture site: including the femoral neck, intertrochanteric, and subtrochanteric. 3. Fracture type: primary and secondary fractures. If there were fragility fractures before this fracture, such as the very common osteoporotic vertebral compression fractures, hip fractures, wrist fractures, and proximal humeral fractures, they were secondary fractures; if not, they were primary fractures. 4. Medical history: whether a patient had been diagnosed with diabetes, HYP, MAL, or KD was obtained from the medical records, which were provided by the patient and/or their family at the time of admission. 5. LD on admission: this can be positive or negative. Various types of pneumonia, tuberculosis, pleural effusion, and/or structural changes, such as pulmonary bullae and fibrosis, are positive but do not include locally stable small lesions, such as stable calcifications. They are determined by chest X-ray or chest CT. Otherwise, they are negative. In particular, this indicator did not include lung tumors, which were classified as MALs. 6. ALI: positive mainly means that the patient could not walk independently or complete the basic activities of daily life without the help of others before the fracture. This could be due to various reasons, including sequelae of cerebrovascular events, severe hip or knee arthritis, Alzheimer's disease, or severe depression. Otherwise, negative. 7. CCD: being positive for this indicator means that the patient had been diagnosed with diseases such as myocardial infarction, cerebral infarction, or cerebral hemorrhage; alternatively, CT and/or MRI examination of the head after admission showed the presence of old local infarct changes. In addition, this also includes intravascular thrombosis as shown by vascular ultrasound examination of the extremities but does not include atherosclerotic plaque formation, stenosis, or other changes. These examinations are not routine examinations after a patient is admitted. If a patient did not undergo these tests and had no history of a diagnosis of the abovementioned cardiovascular and cerebrovascular diseases, they were regarded as negative. 8. PaO_2_, BS, SC, Hb, TP, and ALB: These six indicators are routine laboratory test items after admission. Generally, specimens are collected at 7 o'clock in the morning on the second working day after admission, and all of the results are reported in the afternoon. 9. MAP: MAP is obtained from the one-third systolic blood pressure plus the two-thirds diastolic blood pressure. This value is obtained from the vital signs’ examination of a patient performed immediately after admission. 10. Skeletal traction: in general, skeletal traction was considered on the day of admission. If patients refused or if it was decided to perform surgery, bone traction was not applied. Almost all patients given this treatment underwent tibial tuberosity traction.

Through analysis of the above predictors, we reduced the number of candidate predictors. We excluded medical insurance, fracture site, fracture type, time from fracture to hospitalization, time from hospitalization to operation, and skeletal traction. Regardless of the results of these indicators, they did not affect the choice of surgical treatment. According to the principle of priority of quantitative indicators, diabetes, kidney disease, selected BS, and SC were excluded. Although MAP is a quantitative variable, it is easily affected by various factors and has poor stability, so we chose HYP instead. There is some overlap between serum TP and ALB. Previous studies [9, 10] have shown that low ALB is a prognostic factor for death after fracture, so TP was excluded. Based on the principle of stability, PaO2 was excluded. Because many elderly patients are often given oxygen immediately after admission, it was difficult to strictly follow the doctor’s instructions to stop oxygen inhalation two hours before measuring the index, making it very unstable across patients. As a result, 12 candidate predictors were retained, including age, sex, MLA, LD, ALI, CCD, SUR, BS, SC, Hb, ALB, and HYP.

### Sample size

We did not specifically calculate the required sample size. The reason for this is that the sample size is affected by many factors, and there is no well-known calculation method [11–13]. Second, in reality, we cannot easily expand a single-center study into a multicenter trial or arbitrarily extend the research time to increase the sample size.

According to widely accepted empirical guidelines, we aimed to achieve at least 10 events per variable (EPV) or at least a total of 100 events in the sample. Fortunately, many empirical studies can provide guidance as to how to develop predictive models based on a small sample [14, 15].

### Missing data

**Fig. 2:**
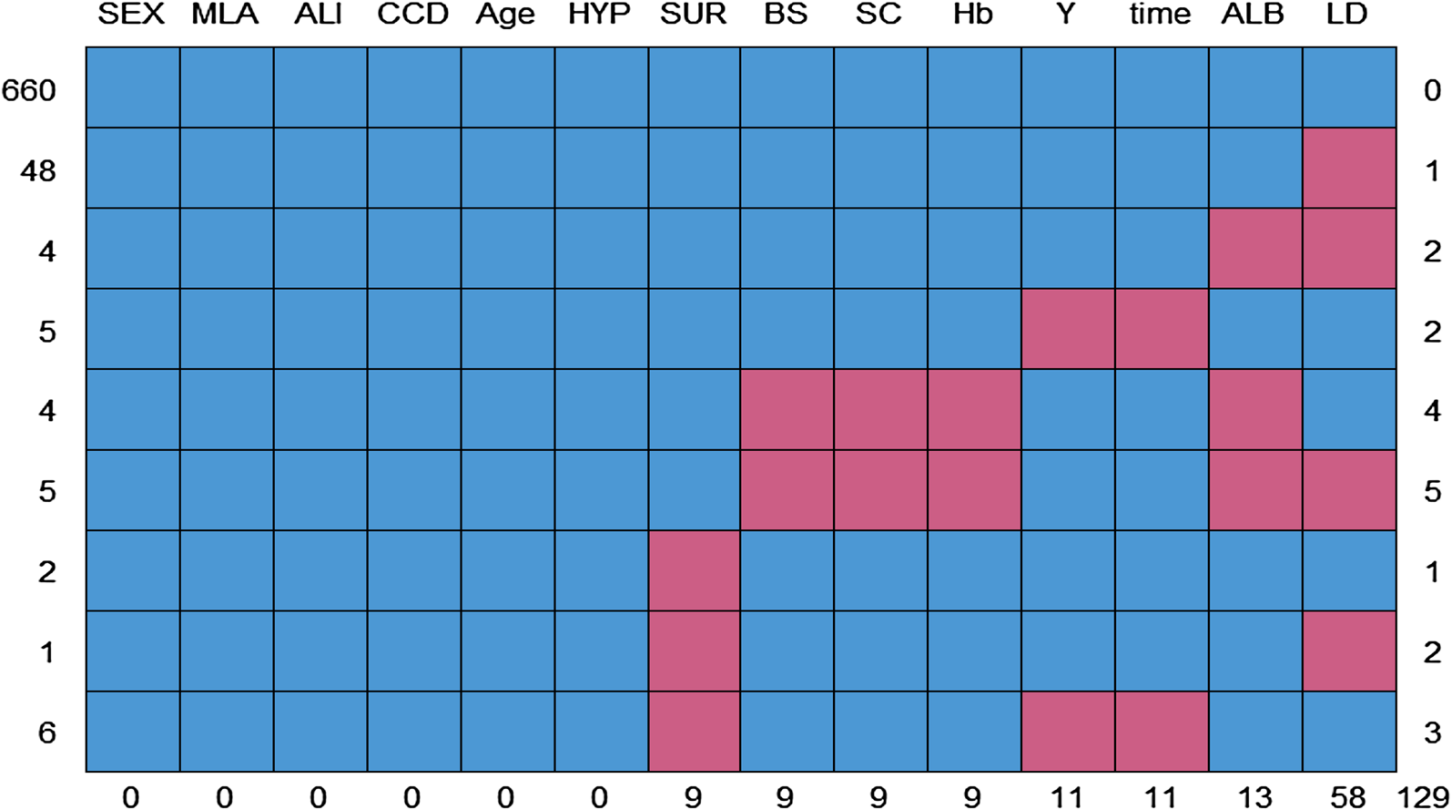
The characteristics of missing data. *Note*: Blue means no missing and red means missing. The label above the graph represents candidate predictors, the below represents the missing number of each candidate predictor, the left represents the number of observations with the same missing pattern, and the right represents the number of types with missing predictors. For instance, ALB predictor has 13 missing values. There are four observations missing both ALB and LD

The characteristics of the missing data are shown in Fig. 2.

Among the 14 variables (two outcome variables, death (Y) and time, and 12 covariates), there were eight variables with missing values, including SUR (containing nine missing values), BS (9), SC (9), Hb (9), ALB (13), LD (58), Y (11), and time (11). A total of 660 observations had no missing data, and five variable values were missing among five observations.

Whether the missing data came from covariates or outcomes, we used the mice package for single imputation (SI) in R software. Although multiple imputation (MI) could be used, the reasons why we chose SI are as follows. First, we had relatively little missing data. Among all of the variables with missing values, LD had the highest missing rate, which was 7.9%, and the missing rates of the remaining variables were less than 2%. Second, an SI dataset can be easily created from the first of a series of MI datasets, and it avoids complicated combinations over multiple MI datasets [16]. Third, no method has been found to combine LASSO models derived from multiple MI datasets [17]. Finally, empirical research has shown that the estimation of model regression coefficients is very consistent between the SI dataset and the MI stacked dataset [18].

Because our SI dataset came from the first of the MI data series, we also analyzed the missing mechanisms of the data and explained the method of generating the MI data series. The missing data were mainly related to the choice of treatment methods for the patients. When patients and their families were not actively involved or were skeptical about surgical treatment, they often refused any routine preoperative preparations. In this case, several variable values were often missing together. Therefore, missing data were often seen among patients who took conservative treatment. This is a "missing at random" (MAR) situation, and MI can effectively address this problem [19–21]. The 12 candidate predictors and the outcome of event and time were all included in the imputation procedure. In this way, eight variables, including LD, SUR, BS, SC, Hb, ALB, Y, and time, were imputed. Among them, the two factor variables, LD and SUR, were imputed using the logistic regression (logreg) method, while the remaining six variables were imputed using the predictive mean matching (pmm) method. No interaction terms were introduced during the MI procedure.

### Statistical analysis methods

The distributions of the five continuous variables including age, BS, SC, Hb and ALB of the 12 candidate predictors were checked for extreme values. After excluding input errors, only SC was found to have obvious extreme values. The extreme values of SC were winsorized to avoid excessive leverage effects. This was done by shifting the values above the 99th centile (eight values, and among them, the maximum was 860 μmol/L) to the truncation points (99th centile, 190.75 μmol/L).

To make full use of the information, we did not categorize any continuous variables, and all kept their original scales. For all continuous variables, linear and nonlinear relationships with the outcome were fitted. The nonlinearity was fitted by using restricted cubic splines (RCSs), and 3, 4, and 5 knots were compared for each variable. In particular, we also checked the log transformation for SC. In addition, we plotted the relationship between the fitted variables and the outcome to check the biological rationality. Based on a higher Waldχ2 value but a lower degree of freedom (df) and biological rationality, the final coding of each number variable in the model was determined (Table 1). The optimal coding for each predictor was as follows: age: linear; BS: nonlinear (RCS, 3 knots); SC: linear; Hb: nonlinear (RCS, 3 knots); ALB: linear.

**Table 1:** Optimal coding exploration for continuous predictors (complete case analysis)

Predictor	Coding	Waldχ^2^	*df*	*p* value
Age	Linear	24.96	1	< .0001
	RCS (3)	22.03	2	< .0001
	RCS (4)	23.90	3	< .0001
	RCS (5)	23.62	4	0.0001
BS	Linear	0.61	1	0.4345
	RCS (3)	0.77	2	0.6798
	RCS (4)	5.66	3	0.1291
	RCS (5)	6.39	4	0.1719
SC	Linear	13.27	1	0.0003
	RCS (3)	12.77	2	0.0017
	RCS (4)	13.94	3	0.0030
	RCS (5)	13.96	4	0.0074
	Log	11.25	1	0.0008
Hb	Linear	10.19	1	0.0014
	RCS (3)	15.14	2	0.0005
	RCS (4)	14.65	3	0.0021
	RCS (5)	15.36	4	0.0040
ALB	Linear	33.97	1	< .0001
	RCS (3)	31.76	2	< .0001
	RCS (4)	31.30	3	< .0001
	RCS (5)	32.07	4	< .0001

According to the principle of simplicity of modeling, all seven categorical variables of SEX, MLA, LD, ALI, CCD, HYP, and

SUR were coded as binary variables.

#### Type of model

Because our goal was a 1-year survival rate, a time-to-event outcome, we chose the Cox proportional hazards model.

#### Predictor selection during modeling

Based on our small sample and empirical studies [14, 15, 22, 23] showing that stepwise selection deteriorates the predictive quality of the model in small datasets, we chose to build a full model that included all 12 candidate predictors. We further refined the full model using the LASSO method, where selection was achieved through shrinking regression coefficients to zero [24, 25]. Therefore, we developed a LASSO model.

#### Interaction terms

To avoid the type I statistical error of multiple repeated detections of interactions [26], we tested the overall interactions of age with the other remaining predictors [27]; the same test was also performed for sex. If there was statistical significance as a whole, then we introduced the statistically significant interaction terms; otherwise, the possibility was excluded. We also tested the proportional hazards assumption.

#### Model performance

Overall performance measures of Nagelkerke’s R^2^ are presented.

The concordance (c) statistic is given as the LASSO model’s discrimination measure, which was further illustrated by dividing the predictions into three groups and plotting the Kaplan–Meier curves of each group.

We did not evaluate the calibration because the “assessment of calibration” makes little sense in the development data, while it is essential for external validation [28].

#### Internal validation

Then, we assessed the internal validity with a bootstrapping procedure of 1 000 repetitions for a realistic estimate of the performance of the LASSO and full model in similar future patients.

## Results

### Participants

**Fig. 3:**
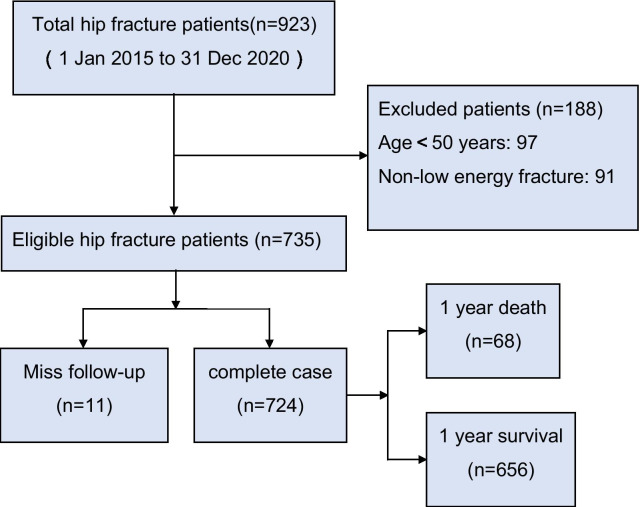
Participant flow diagram

From January 2015 to December 2020, 923 patients with hip fractures were admitted to our hospital, and 735 patients were included in this study. As of the end of the last interview, May 28, 2021, 11 patients were lost to follow-up, and the average follow-up time was 325.6 days per person. The selection process and specific characteristics of the sample are shown in Fig. 3 and Table 2, respectively.

**Table 2:** Participant characteristics

Characteristic	Missing values, *n (%)*	Value
**General characteristics**		
Mean age (years)	0	74.8(SD, 9.5) (range 50–103)
Male	0	279 (38.0%)
Medical insurance	0	
Employee medical insurance (EMI)		101 (13.7%)
Non-EMI		634 (86.3%)
**Fracture-related**		
Fracture site	0	
Femoral neck		305 (41.5%)
Intertrochanteric		413 (56.2%)
Subtrochanteric		17 (2.3%)
Fracture type	0	
Primary		689 (93.7%)
Secondary		46 (6.3%)
Fracture to admitted (d)	0	2.3 (SD, 5.9) (range 0–62)
Admitted to surgery (d) (637patients) #	0	5.5 (SD, 3.3) (range 1–45)
Length of stay (LOS)	0	13.0 (SD, 6.4) (range 1–52)
**Medical history**		
Diabetes	0	116 (15.8%)
Hypertension (HYP)	0	357 (48.6%)
Malignancy (MLA)	0	18 (2.4%)
Kidney disease (KD)	0	11 (1.5%)
Lung disease (LD)	58 (7.9%)	271 (36.9%)
Ability of living independence (ALI) (no = 0, yes = 1)	0	107 (14.6%) (no = 0)
Cardiovascular and cerebrovascular diseases (CCD)	0	295 (40.1%)
**Clinical indicators**		
Blood sugar (BS)(mmol/L)	9 (1.2%)	6.8 (SD 2.4) (range 3.4–21.6)
Serum creatinine (SC) (μmol/L)*	9 (1.2%)	70.3 (SD, 23.8) (range 26.0–190.8)
Hemoglobin (Hb)(g/L)	9 (1.2%)	117.8 (SD, 20.1) (range 43–179)
Albumin (ALB)(g/L)	13 (1.8%)	37.9 (SD, 4.1) (range 21.8–48.2)
Mean arterial pressure (MAP) (mmHg)	0	104.8 (SD, 16.2) (range 56–182)
Partial pressure of oxygen (PaO_2_) (mmHg)	212 (28.8%)	75.4 (SD, 17.4) (range 33–176)
Total protein (TP) (g/L)	16 (2.2%)	63.4 (SD, 6.8) (range 44.6–105.0)
**Treatment**		
Skeletal traction	0	375 (51.0%)
Surgery	9 (1.2%)	637 (86.7%)

*SD*: standard deviation

*Means the SC value was winsorized; before this, the mean was 72.14 μmol/L (SD, 42.6) and the range was [26.0, 860.0]

^#^Means it includes only 637 patients who were operated on in our hospital

### Model development

By performing SI on the raw data, we finally obtained a no missing value sample with 735 participants and 68 interesting events. The EPV ratio = 68/12 = 5.7. Admittedly, it was a small sample [15]. Based on this sample, all 12 candidate predictors were used to develop a full model. We also examined the unadjusted association between each predictor and the outcome (Table 3).

**Table 3:** Association between each predictor and outcome from the SI dataset

Characteristic	Patients with an outcome (n = 68)	Patients without an outcome (n = 667)	Univariate hazard ratios (95% *CI*)	Full model hazard ratios(95%*CI*)	LASSO model hazard ratios(95%*CI*)
**Demographic characteristics**					
Age (years) (**82 vs. 68**)	80 (*SD* 7.9)	74 (*SD* 9.5)	2.7 (1.8, 4.0)	2.2 (1.4, 3.5)	1.8 (1.2, 2.8)
Sex (Female = 0, Male = 1, **1 vs. 0**)	35 (51.5%) (Male)	244 (36.6%) (Male)	1.7 (1.1, 2.8)	1.7 (1.0, 2.8)	1.4 (0.8, 2.2)
**Medical history**					
MLA (No = 0, Yes = 1, **1 vs. 0**)	5 (7.4%) (Yes)	13 (1.9%) (Yes)	3.2 (1.3, 7.9)	4.1 (1.4, 11.9)	2.7 (1.0, 7.7)
LD (No = 0, Yes = 1, **1 vs. 0**)	34 (50.0%) (Yes)	266 (39.9%) (Yes)	1.5 (1.0, 2.5)	1.0 (0.6, 1.7)	-
ALI (No = 0, Yes = 1, **0 vs. 1**)	15 (22.1%) (No)	92 (13.8%) (No)	1.7 (0.9, 3.0)	1.5 (0.8, 2.9)	1.3 (0.7, 2.5)
CCD (No = 0, Yes = 1, **1 vs. 0**)	37 (54.4%) (Yes)	258 (38.7%) (Yes)	1.8 (1.1, 3.0)	1.5 (0.9, 2.6)	1.4 (0.8, 2.4)
HYP (No = 0, Yes = 1, **1 vs. 0**)	34 (50.0%) (Yes)	323 (48.4%) (Yes)	1.1 (0.7, 1.7)	1.5 (0.8, 2.5)	1.2 (0.7, 2.0)
**Clinical indicators**					
BS (mmol/L) (**7.1 vs. 5.5**)	6.9 (*SD* 2.7)	6.8 (*SD* 2.3)	1.0 (0.9, 1.2)	1.1 (0.9, 1.2)	–
SC (μmol/L) (**79 vs. 56**)	80.1 (*SD* 32.2)	69.2 (*SD* 22.5)	1.3 (1.1, 1.6)	1.2 (1.0, 1.5)	1.2 (1.0, 1.4)
ALB(g/L) (**40.8 vs. 35.3**)	35.1 (*SD* 4.3)	38.1 (*SD* 4.0)	0.4 (0.3, 0.6)	0.6 (0.4, 0.8)	0.6 (0.5, 0.9)
Hb(g/L) (**132 vs. 104**)	111.2 (*SD* 23.3)	118.4 (*SD* 19.6)	0.6 (0.5, 0.9)	1.1 (0.7, 1.5)	–
**Treatment**					
SUR (No = 0, Yes = 1, **0 vs. 1**)	30 (44.1%) (No)	59 (8.8%) (No)	6.9 (4.3, 11.2)	5.2 (3.1, 8.7)	4.8 (2.9, 8.0)

*SD*: standard deviation; *CI*: confidence interval

### Model specification

In exploring the optimal coding of five continuous variables, we found that it was suitable for BS and Hb adopting nonlinearity (both RCS, 3 knots), while age, SC, and ALB maintained linearity. However, we all adopted linear coding when modeling because: 1. In the full model coding BS and Hb as nonlinear, the nonlinear Wald statistics had no statistical significance, with P values of 0.834 and 0.088, respectively. Compared with the full linear model (total Chi-square 82.01, 12 df), for the nonlinear model (total Chi-square 85.23, 14 df) the total Wald χ2 value only increased by 3.22 with more the 2 df. 2. “In smaller datasets, we may simply have to rely on the additivity assumption to be reasonable” [29]. Nonlinear coding makes the model complex and difficult to interpret, which violates the principle of simplicity. 3. Using the explained variation by R^2^, we can compare the effects of different encodings of predictors on the model [28]. Compared with the linear full model (R^2^ = 0.179), the R^2^ of the nonlinear full model (R^2^ = 0.184) only increased by 0.005. In view of the above results, it is reasonable to use nonlinear coding for BS and Hb.

Neither age nor sex had a significant total interaction with the remaining variables; their *p* values were 0.343 and 0.305, respectively. Therefore, no interaction terms were introduced in the full model. The coefficients of the full model and the LASSO model, as well as the encoding forms of each variable, are given in Table 4. The overall test for the proportional hazards assumption of the full model was not statistically significant (overall χ^2^ 14.1975, 12 df, p = 0.288), with a nonproportionality suggested for LD.

**Table 4:** Presenting the model, including the baseline survival, for 1-year survival (from the SI dataset)

Predictors	The full model			The LASSO model		
	β coefficient	SE	*p* value	β coefficient	SE	*p* value
Age (linear)	0.0567	0.0167	0.0007	0.0421	0.0156	0.0070
Sex = 1 (Female = 0, Male = 1)	0.5125	0.2591	0.0479	0.3047	0.2567	0.2352
MLA = 1 (No = 0, Yes = 1)	1.4137	0.5430	0.0092	1.0000	0.5333	0.0608
LD = 1 (No = 0, Yes = 1)	– 0.0083	0.2625	0.9746	–	–	–
ALI = 1 (No = 0, Yes = 1)	– 0.4368	0.3268	0.1813	– 0.2721	0.3253	0.4028
CCD = 1 (No = 0, Yes = 1)	0.4075	0.2781	0.1428	0.3432	0.2754	0.2126
HYP = 1 (No = 0, Yes = 1)	0.3837	0.2807	0.1716	0.1713	0.2743	0.5322
BS (linear)	0.0350	0.0448	0.4345	–	–	–
SC (linear)	0.0091	0.0041	0.0284	0.0080	0.0041	0.0502
ALB (linear)	– 0.1001	0.0336	0.0029	– 0.0819	0.0310	0.0082
Hb (linear)	0.0020	0.0067	0.7659	–	–	–
SUR = 1 (No = 0, Yes = 1)	– 1.6523	0.2592	< 0.0001	– 1.5734	0.2546	< 0.0001

S_0_ = 0.984 (1-year baseline survival)

“–” means that this variable was removed during the LASSO shrinking process

**Fig. 4:**
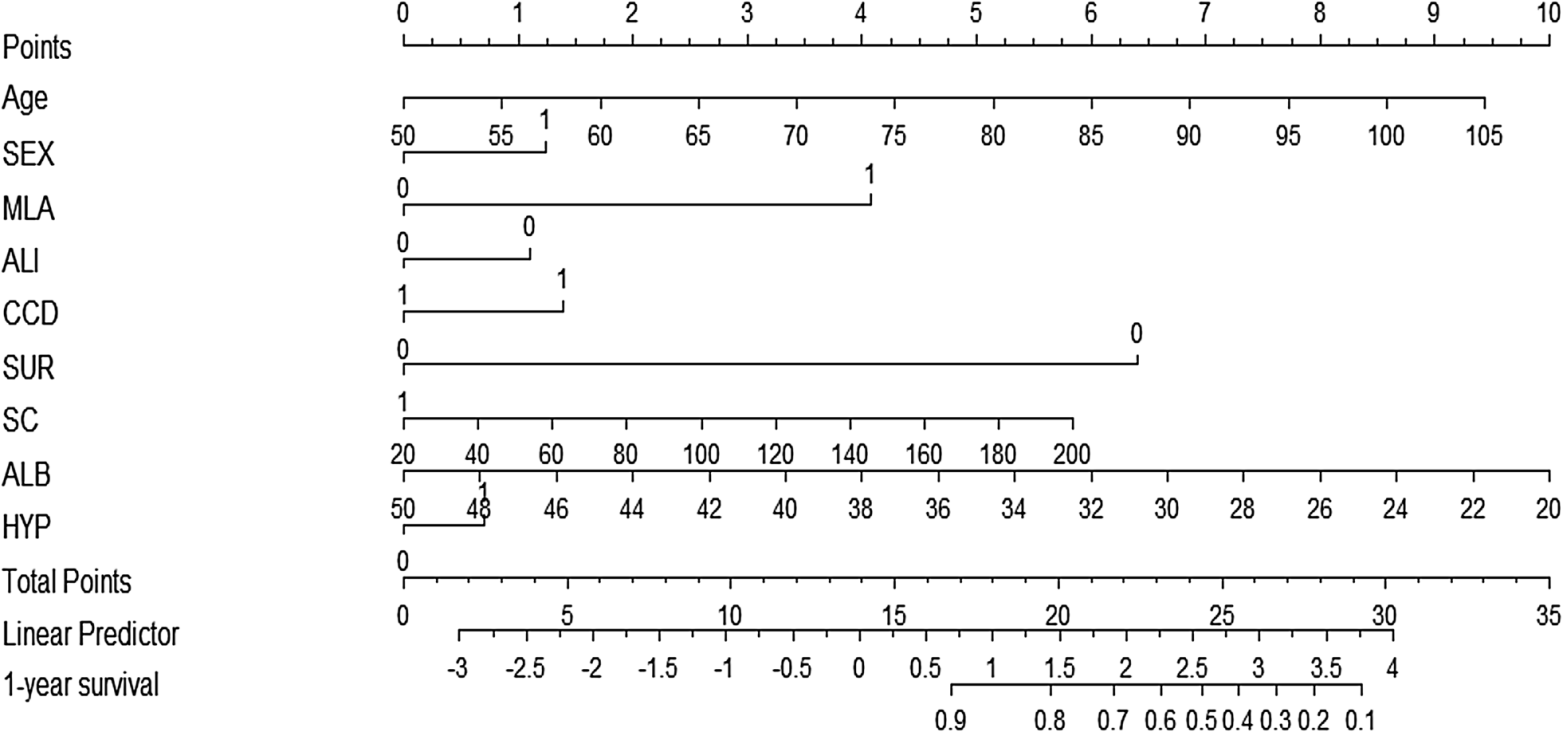
Nomogram based on the LASSO model. *Note*: For example, a man (SEX = 1.3) with fragility hip fracture, 80 years old (age = 5.1), no malignant tumor (MLA = 0), self-care ability well (ALI = 0), having cerebral infarction history (CCD = 1.4) and no hypertension (HYP = 0), serum creatinine 100 μmol/L (S C = 2.6), albumin 42 g/L ( ALB = 2.7), and choosing conservative treatment (SUR = 6.4), so the total score = 19.5, the 1-year survival close to 0.8 or 80%. The corresponding 1-year mortality is 20%

Based on the LASSO model, we created a monogram so that the readers can easily apply this model to similar patients (Fig. 4 and Table 5).

**Table 5:** A simple scoring system for calculating the survival/mortality

Predictor	Variable range	Score range	Total score	Survival	Mortality
Age (years)	50–105	0–9.4	15.4	0.9	0.1
Sex (Female = 0, Male = 1)	0	0	20	0.8	0.2
	1	1.3	22	0.7	0.3
MLA (No = 0, Yes = 1)	0	0	23	0.6	0.4
	1	4	24.3	0.5	0.5
ALI (No = 0, Yes = 1)	0	1.1	25.5	0.4	0.6
	1	0	26.6	0.3	0.7
CCD (No = 0, Yes = 1)	0	0	27.8	0.2	0.8
	1	1.4	29.2	0.1	0.9
SUR (No = 0, Yes = 1)	0	6.4			
	1	0			
SC (μmol/L)	20–200	0–5.8			
ALB (g/L)	20–50	10–0			
HYP (No = 0, Yes = 1)	0	0			
	1	0.7			

The predicted survival probability of the patient with fragility hip fracture within 1 year after fracture is calculated by p = 0.984^exp (0.042*Age+0.305*SEX+MLA−0.272*ALI+0.343*CCD−1.573*SUR+0.008*SC−0.082*ALB+0.171*HYP)^; the corresponding mortality rate is 1-p

### Model performance

**Fig. 5:**
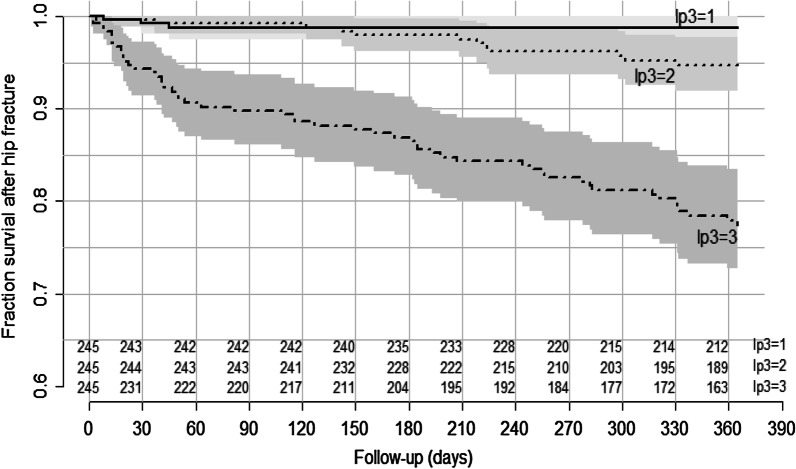
Kaplan–Meier curves for three risk groups of low, middle, and high

The c statistic of the final LASSO model was 0.814 (95% CI 0.762–0.865). Discrimination was further illustrated by dividing the predictions into three groups, low, middle and high and plotting the Kaplan–Meier curves of each group (Fig. 5). We found that patients in the high-risk group (lp3) had a considerably worse chance of survival at 1 year after hip fracture: approximately 77%. In addition, we also presented the overall performance measures R^2^ and the optimism gained through internal validation of 1000 bootstrap repetitions so that readers can understand the predictive effectiveness of the model for similar patients. The same performance measures for the full model are also presented (Table 6).

**Table 6:** Model performance measures

Performance measures	Full model			LASSO model		
	Original	Internal validation (B = 1000)	Optimism	Original	Internal validation (B = 1000)	Optimism
C	0.816	0.790	0.026	0.814	0.795	0.019
R^2^	0.188	0.147	0.041	0.187	0.158	0.030

## Discussion

### Limitations

This study has the following limitations. First, the worst shortcoming is the low effective sample size where EPV is less than 6 (5.7 events per variable) and the total interesting events are less than 100. This affects the stability of the LASSO model and usually leads to overfitting and optimism, although we tried to compensate for these shortcomings with the methodology. Second, because the LASSO models derived from each of the MI datasets have not been easily synthesized in an efficient way, the MI was not used for the missing data. This made the estimation of the model fail to fully consider the uncertainty of the inference of missing values. Third, to increase the EPV ratio, the number of candidate predictors was reduced as much as possible. This resulted in other indicators related to survival reported by other studies, such as the time to surgery [30–35], time from the trauma to operation [36, 37], the patient financial situation [38], fracture sites [39, 40], and fracture season [41] not being included in the model, not to mention other possible related factors, such as muscle loss [42] and multidisciplinary management [43, 44]. The calibrated R^2^ (0.158) also confirms that there is still much variability that cannot be explained by the model. Fourth, the design of this study was a retrospective cohort study. Compared with an ideal prospective cohort study, the collection of various values cannot be controlled in advance. This results in the accuracy of the data being somewhat inadequate, thus affecting the credibility of the model prediction. Fifth, the representativeness of the sample is insufficient. Most of the patients in this sample were rural patients (approximately 80% of the sample, but the urban population was approximately 64% in 2021 in China), which might affect the applicability and generalizability of the model. Finally, this study only performed internal validation. Although the results show that the performance of the model is acceptable, in view of the abovementioned shortcomings, external validation is obviously necessary.

### Interpretation

Unlike the models of Elliott 2003 [5] and Anita 2009 [4], we avoided using various scoring models as predictors to keep the model simple and easy for clinical use. Different from the Maxwell 2008 [6] model, we avoided converting numerical variables into categorical variables on the basis of ensuring the stability of variable values to improve the utilization of valuable data. We used very common preoperative variables that can be quickly obtained after hospitalization to predict their 1-year survival. Our final model has no nonlinear coding or interaction terms and only contains nine covariates, including age, sex, a history of malignant tumors, self-care ability before fracture, history of cardiovascular and cerebrovascular diseases, history of hypertension, whether to choose surgery, and blood creatinine value and albumin value at admission. Therefore, we have achieved the goal of developing a simple model to guide doctors and patients to rationally choose treatment methods. Admittedly, whether the predictive model is practical needs subsequent clinical feedback, but its simplicity has laid a good foundation for practicality.

**Fig. 6:**
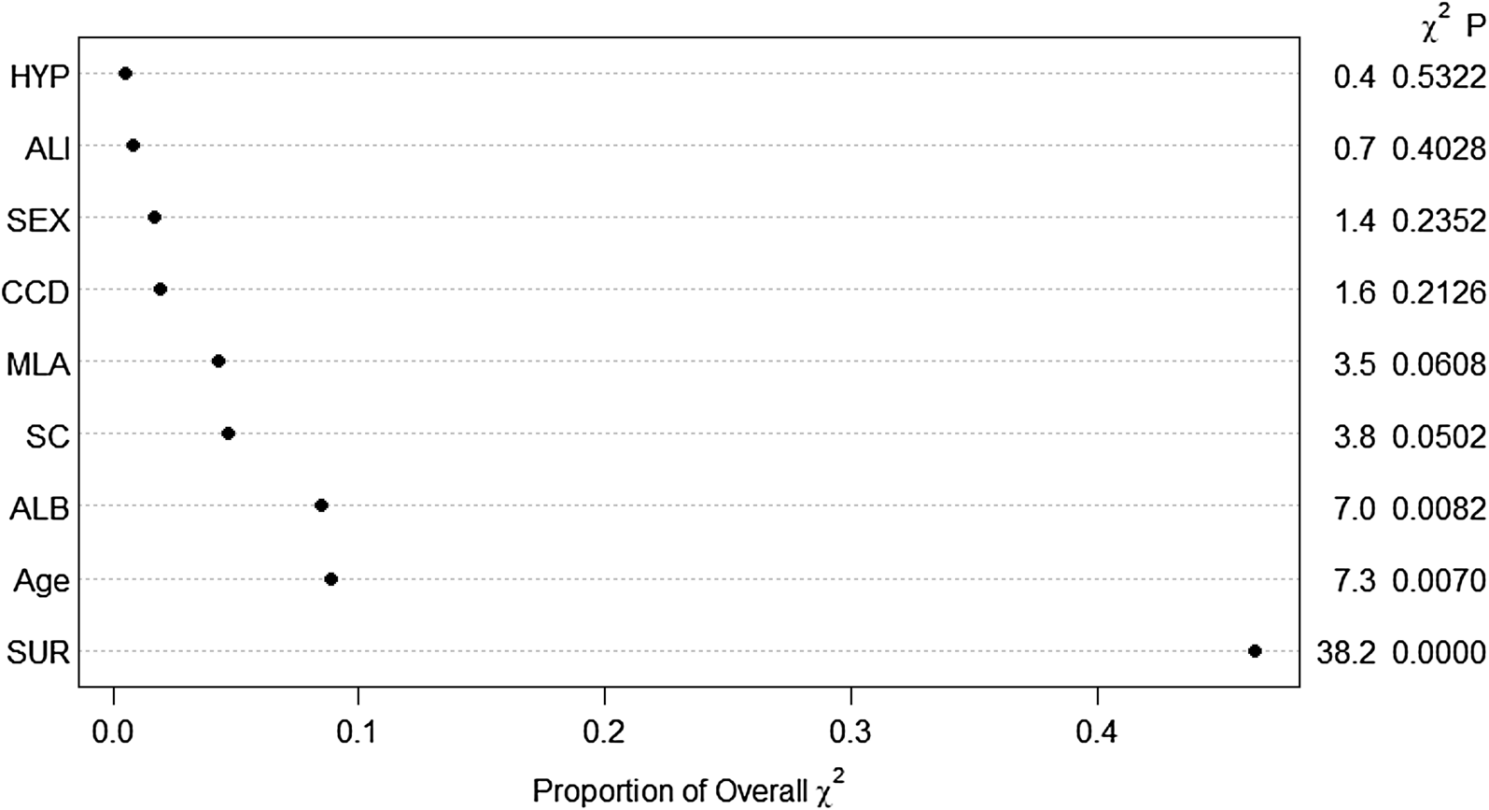
Relative contribution of each predictor to the full LASSO prediction model

This may be the first survival prognosis model for fragile hip fracture developed for Chinese subjects. The multivariate regression analysis of our LASSO model showed that age, serum albumin value at admission, and whether to choose surgery were effective predictors of the 1-year survival rate, consistent with the results of many studies (Fig. 6) [9, 31, 34, 45–47]. In the full model without shrinkage of the coefficients, in addition to the above three predictors, sex, history of malignant tumors, and creatinine value at admission were also valid predictors. The prognostic validity of these three variables has been reported by practically all studies [31, 34, 39, 45, 46, 48–50]. This difference between the two models stems from the fact that the effect strength between the latter three predictors and the outcome is weaker than that of the first three. The patient's blood pressure, blood sugar, hemoglobin, lung disease, self-care ability before fracture, and history of cardiovascular and cerebrovascular disease were not significantly related to the 1-year survival rate. However, many studies [33, 37, 46, 49, 51–53] have shown that lung disease and preoperative walking ability are related to the postoperative mortality of hip fractures. The reasons for this difference may include the following: The study population was different since most of the research subjects were limited to patients treated by surgery; the age range was different, most of whom were patients over 65 years; and the race of the participants was different.

The discrimination c value for the final LASSO model was 0.814 (95% CI 0.762–0.865), located in an intermediate accuracy range (0.71–0.90), so the discriminative ability of the model was acceptable. After correcting the optimism through internal validation, the c value was reduced from 0.814 to 0.795, a decrease of 0.019, which is still acceptable. Similar results are also seen in the full model performance evaluation. In contrast to the full model, the LASSO model decreased in optimism (from 0.026 to 0.019). These results prove that it is reasonable to use the LASSO method for model estimation based on the full model under the condition of a small sample. Similar results are also seen in the overall performance measure R^2^. This paper is only a prediction model development study, so we can only obtain the internal validity (or “reproducibility”) (the discriminative ability index c statistic was 0.795, close to the "good" level) of the model applied to potential populations through internal validation technology [54]. For the more concerned external validity (or “generalizability”/ “transportability”) applied to reasonably related populations, this needs to be obtained from the following external validation researches [55].

### Implications

The applicable objects of this model are patients with fragile hip fractures. Obviously, it is not applicable to patients with nonfragility fractures. Because this model is simple and clear, in addition to orthopedic surgeons, patients and their family members can also easily grasp the model. The optimal application environment is general hospitals with nonurban populations as the main patients. The next external validation should be a general hospital with an urban population. Limited by sample size, this model does not consider many variables. It is recommended to include more relevant predictors in future model update studies.

## Conclusion

Using the SFHF model, doctors can quickly classify patients with fragile hip fractures into low-, medium-, and high-risk patients. On this basis, the treatment plan can be more reasonably determined by the doctors, patients, and their families. When a patient is assessed as high risk, and surgery may not effectively improve the survival rate, conservative treatment may be more beneficial. It is believed that the use of this model will have a positive effect on avoiding doctor–patient conflicts.

## Data Availability

The datasets used during the current study are available from the corresponding author on reasonable request.

## Abbreviations

LASSO: Least absolute shrinkage and selection operator;
SFHF: Survival in patients with fragile hip fracture
SUR: Surgery
ALB: Albumin
SC: Serum creatinine
MAL: Malignancy
HYP: Hypertension
ALI: Ability to live independently
CCD: Cardiovascular and cerebrovascular diseases
LOS: Length of stay
PaO_2_: Partial pressure of oxygen
TP: Total protein
EPV: Events per variable
Y: Death
SI: Single imputation
MI: Multiple imputation
RCS: Restricted cubic splines
df: Degrees of freedom
SD: Standard deviation
CI: Confidence interval
